# Relational Stability: A New Strategy for Defining the Human Core Microbiome

**DOI:** 10.1007/s43657-025-00236-7

**Published:** 2025-03-20

**Authors:** Liping Zhao

**Affiliations:** Department of Biochemistry and Microbiology, Rutgers Center for Microbiome Analysis, Rutgers University, New Brunswick, 08901 USA

## Core Microbiome: The Hidden Organ Essential for Human Health

The core microbiome, often described as a ‘hidden organ’, has captivated researchers as a fundamental key to understanding the intimate relationship between microbial ecosystems and human health (Turnbaugh and Gordon 2009). This specialized subset of microbial entities, ever-present across populations, transcends the variations of genetics, diet, and lifestyle to act as a stabilizing force in the gut. By orchestrating the gut microbiome’s structural integrity and ecological balance, these microbial allies have long ensured human survival, adaptation, and resilience against disease (Shade and Handelsman 2012). From shaping human evolution to revolutionizing precision medicine, the core microbiome stands at the intersection of biology and health, offering profound insights into what keeps us alive and thriving.

Defining the core microbiome is essential for establishing a robust framework for studying the microbiome’s role in human biology (Li et al. 2013). Unlike transient microbial populations that exhibit significant variability across individuals and time, core microbiome members provide a stable reference point for investigating how gut microbes regulate critical physiological processes such as digestion, immune modulation, and metabolic homeostasis. This reference point is crucial for identifying microbial markers that serve as reliable indicators of health and disease, creating opportunities for breakthroughs in developing predictive models for early disease diagnosis.

Furthermore, a well-defined core microbiome advances the development of microbiome-targeted therapies (Zhao 2013). By identifying microbes and their functional traits critical for maintaining health, researchers can design interventions to foster beneficial microbial entities or suppress pathogenic ones. Such targeted approaches hold transformative potential for treating chronic diseases, including obesity, diabetes, inflammatory bowel disease, neurodegenerative disorders, and cancers. These therapeutic innovations derive from an understanding that core microbiome members are the ecosystem’s functional linchpins.

The core microbiome also underpins predictive modeling in personalized medicine. By integrating microbial data with host genomic and metabolic profiles, researchers can develop algorithms to predict individual susceptibility to diseases and responses to treatments. These models enable the customization of interventions, optimizing health outcomes and fulfilling the promise of precision medicine (Neu et al. 2021).

In essence, the core microbiome transcends theoretical interest; it is a practical tool for bridging microbiome science with health care applications. However, achieving a universally accepted definition of the core microbiome remains a formidable challenge (Neu et al. 2021; Shade and Handelsman 2012). Conventional approaches rely on cataloging microbial taxa that are commonly shared across populations. This taxonomic overlap assumes that shared presence equates to importance, yet this assumption often falls short. Core microbiome members are indeed commonly shared, but not all commonly shared microbes contribute significantly to critical ecosystem functions.

This limitation is evident when considering the functional redundancy and strain-specific diversity inherent in microbial communities. Taxonomic labels often fail to capture the ecological and functional roles of individual strains, risking the exclusion of key microbial interactions that sustain host health (Zhao et al. 2024; Wu et al. 2021). Consequently, a taxon-based approach to defining the core microbiome may obscure the true contributors to ecosystem stability and function.

## Systems Biology: Stable Relationships Signify Core Components

To address these challenges, we turn to systems biology, which offers a holistic framework for understanding complex adaptive systems (Levin 1998). In such systems, stable relationships—not individual components—signify the core structure. Stable relationships arise from persistent interactions among agents that collectively drive system behavior. For the gut microbiome, this implies identifying microbial relationships that endure across varying conditions, such as dietary interventions or disease states. The principle that “stable relationships signify core components” shifts the focus from cataloging individual taxa to exploring the ecological and functional interdependencies among microbes. This systems-level perspective aligns with the dynamic nature of microbial ecosystems, where resilience and functionality emerge from interconnected networks rather than isolated taxa.

Building on this principle, we propose a novel strategy for defining the core microbiome: identifying stably connected genome pairs across diverse conditions. This approach involves constructing co-abundance networks from microbiome data and searching for genome pairs that exhibit stable correlations under conditions such as dietary interventions or disease states (Wu et al. 2024). Stable genome pairs reflect ecological interactions, including cooperation, competition, and niche sharing, which are essential for maintaining ecosystem integrity.

Our analysis of 38 microbiome datasets spanning dietary interventions and 15 diseases revealed a consistent Two Competing Guilds (TCG) structure as a core signature. The TCG model comprises the Foundation Guild (FG), dominated by Short Chain Fatty Acids (SCFA)-producing bacteria, and the Pathobiont Guild (PG), enriched with opportunistic pathogens and pro-inflammatory microbes. These guilds represent opposing functional forces within the microbiome, balancing health-promoting and disease-driving dynamics. TCG members are the most stably and widely connected in the ecosystem network: 85% of ecological interactions center around them, though they constitute less than 10% of total members. Removing FG or PG members disrupts the network’s integrity, underscoring their foundational role as the backbone of the gut microbial ecosystem (Wu et al. 2024).

The TCG model elucidates mechanisms underlying disease progression and recovery (Zhao et al. 2018). FG members produce SCFAs, such as acetic and butyric acids, which inhibit PG microbes, enhance gut barrier integrity, mitigate inflammation, alleviate insulin resistance, and promote satiety hormone production (Wu et al. 2024; Zhao et al. 2018). Conversely, PG members produce endotoxins, indole, and hydrogen sulfide, which drive inflammation and disrupt metabolic homeostasis. The stable relational dynamics between FG and PG determine the microbiome’s ecological balance, directly influencing host health outcomes.

The predictive utility of stable relationships becomes evident in Artificial Intelligence (AI)-based modeling (Wu et al. 2024). Using stably connected genomes in TCGs as features significantly improved the classification of disease versus control samples and enhanced the prediction of treatment outcomes compared to models relying on taxonomic composition. These findings underscore the value of focusing on stable relationships for advancing precision medicine and microbiome science.

## Evolutionary Perspective of Relational Stability

The concept of relational stability is grounded in the idea that core microbiome members maintain consistent functional relationships within the gut ecosystem across diverse conditions. From an evolutionary perspective, these stable relationships are not incidental but represent the result of millennia of co-evolution between humans and their microbiota. Core microbiome members, such as *Faecalibacterium prausnitzii* and *Roseburia spp.*, have thrived as cooperative partners, performing indispensable functions such as fermenting dietary fibers into SCFAs, reducing systemic inflammation, and fortifying gut barrier integrity (Lee et al. 2024; Zhao et al. 2018; Tazoe et al. 2008; Wong et al. 2006). These contributions exemplify the persistence of functional interactions between host and microbiome under a variety of historical dietary and environmental conditions.

However, the abrupt dietary shifts brought by agricultural and industrial revolutions tested the resilience of these co-evolved relationships (Cano et al. 2014; Konner and Eaton 2010; Eaton and Konner 1985). Modern diets, characterized by refined carbohydrates and low fiber, have disrupted the ecological stability of the gut, favoring transient and pathogenic microbes over core members. Despite this disruption, the principle of relational stability holds: core microbiome members, by definition, are those that retain their ecological and functional roles even in the face of such perturbations. For example, studies have shown that SCFA-producing bacteria remain functionally connected to metabolic health despite modern dietary pressures, though their ecological dominance diminishes without intervention (Zhao et al. 2018).

This evolutionary perspective reinforces the idea that relational stability is not merely a snapshot of current microbiome function but a reflection of enduring interactions that have withstood environmental changes over time. By focusing on stable relationships, the relational stability framework identifies core microbiome members as those that consistently contribute to ecosystem resilience, even under modern challenges (Wu et al. 2024). This approach allows us to redefine the human core microbiome not by taxonomic ubiquity but by the durability and relevance of functional interactions over evolutionary and environmental timescales.

## The Implications of Relational Stability in Microbiome Research and Beyond

In a broader context, relational stability provides a universal strategy for identifying and understanding the core structures within complex adaptive systems, ranging from natural ecosystems to human social networks. These systems are characterized by intricate networks of agents—such as species, individuals, or entities—whose interactions drive collective functionality and emergent behaviors. By focusing on stable relationships, researchers can pinpoint the persistent interactions that form the backbone of these systems, enabling deeper insights into their resilience, adaptability, and long-term dynamics. For example, in ecological networks, relational stability highlights the keystone species and mutualistic partnerships critical for ecosystem balance (Vitali et al. 2022). In social networks, it reveals enduring interpersonal or organizational connections that sustain community cohesion and functionality (van den Bos et al. 2018).

Relational stability also provides a powerful framework for uncovering principles that govern stability, functionality, and evolution in these systems. Stable relationships often signify foundational roles, whether in maintaining the structural integrity of an ecosystem, driving the robustness of a social group, or supporting the stability of economic systems. By identifying these core interactions, researchers can explore how such relationships facilitate adaptation to external stressors, promote systemic robustness, and contribute to evolutionary success. This approach not only enhances our understanding of the systems in question but also informs strategies to support or restore their balance when disrupted, such as conserving ecological habitats or reinforcing social networks in times of crisis (Wu et al. 2024; Zhao et al. 2018; Zhang et al. 2015; Xiao et al. 2014).

Moreover, the concept of relational stability extends seamlessly into the realm of AI modeling. Stable interactions within complex systems offer a unique opportunity to develop robust and interpretable input features for AI frameworks. The Stably Connected Agent Network (SCAN) AI modeling framework capitalizes on this principle, using stably connected agents—whether microbial genomes, ecological species, or social nodes—as key features for prediction and analysis. In medical applications, SCAN can improve the precision and accuracy of models for diagnosing diseases or predicting treatment outcomes by leveraging stable microbial relationships as indicators of health or disease states. Beyond medicine, SCAN offers transformative potential in diverse fields, from optimizing supply chain networks to improving social policy interventions by modeling stable relational dynamics. This approach underscores the universal applicability of relational stability, providing a foundational tool for advancing research, innovation, and real-world problem-solving across disciplines.

## Conclusion

Relational stability redefines our understanding of the core microbiome, offering a transformative lens through which to study and apply microbiome science. By emphasizing stable relationships as the foundation of the gut microbial ecosystem, this approach moves beyond the limitations of taxonomic overlap, capturing the dynamic and functional interdependencies that sustain host health. The Two Competing Guilds (TCG) model exemplifies this framework, highlighting the ecological balance between health-promoting and disease-driving microbe (Wu et al. 2024; Zhao et al. 2018; Zhang et al. 2015; Xiao et al. 2014). Its applications, from precision medicine to AI modeling, underscore the potential of relational stability to reshape research and interventions across disciplines.

However, the promise of relational stability is only beginning to be realized. Researchers must now embrace this paradigm shift, applying relational stability not only to microbiome studies but also to broader complex adaptive systems. This requires collaboration across fields, integrating insights from ecology, systems biology, and computational science to refine methodologies and expand applications. For microbiome researchers, this means prioritizing the identification of stably connected genome pairs and using these relationships to inform biomarker discovery, therapeutic design, and predictive modeling.

Relational stability offers more than a theoretical framework; it is a practical tool for understanding and restoring balance in systems under stress, from disrupted microbiomes to fragmented ecosystems. By adopting relational stability as a guiding principle, researchers can uncover universal patterns of resilience and functionality, driving innovation and improving outcomes in health care, technology, and beyond. The time to act is now—by focusing on stable relationships, we open new doors to understanding the intricate connections that sustain life and its remarkable adaptability.

## Data Availability

Not applicable.
